# Technetium-99m-marked sentinel lymph node biopsy for oral squamous cell carcinoma as a safe and effective procedure for patient and staff

**DOI:** 10.3389/fonc.2026.1834526

**Published:** 2026-06-29

**Authors:** Christian Soemmer, Felix Bärenfänger, Evangelos Vitkos, Ákos Bicsák, Alicia König, Stefan Hassfeld, Lars Bonitz, Monia Hamami-Arlinghaus

**Affiliations:** 1 Health Faculty, University of Witten/Herdecke, Witten, Germany; 2 Department of Oral and Maxillofacial Surgery, Regional Plastic Surgery, Dortmund General Hospital, Dortmund, Germany; 3 Department of Nuclear Medicine, Dortmund General Hospital, Dortmund, Germany

**Keywords:** biopsy, head and neck neoplasms, lymphatic metastasis, sentinel lymph node, technetium (Tc)-99m

## Abstract

**Introduction:**

Sentinel lymph node biopsy (SLNB) using [99mTc]Tc-albumin nanocolloid has emerged as a minimally invasive staging technique for selected patients with oral squamous cell carcinoma (OSCC). This study evaluates the feasibility of SLNB, the quality of patient selection, and radiation exposure during the procedure.

**Materials and methods:**

This retrospective single-center study included patients with oral squamous cell carcinoma clinically staged as cT1-cT2 cN0 who underwent SLNB using [99mTc]Tc-albumin nanocolloid. Sentinel lymph nodes were identified by preoperative imaging and intraoperative gamma probe detection. Histopathological findings guided further treatment. Radiation exposure to the surgeon was estimated using a point-source approximation. Clinical, pathological, and follow-up data were analyzed descriptively.

**Results:**

A total of 16 patients were included (11 males, 5 females; mean age 70.9 ± 11.1 years). Occult lymph node metastases were detected in 3 of 16 patients (18.8%), while tumor upstaging (pT>cT) occurred in 1 patient (6.3%). Sentinel lymph nodes were successfully identified in all patients. The mean administered technetium-99m activity was 100.9 ± 2.1 MBq, and surgery was performed after a mean interval of 26 h 03 min. The mean duration of surgery was 111.5 min. During surgery, the remaining radioactive activity averaged 5.1 MBq, and the surgeon’s mean radiation exposure was 2.3 μSv. During follow-up, no regional nodal recurrences or delayed nodal failures were observed, and all patients were alive at the time of database lock.

**Conclusion:**

Sentinel lymph node biopsy may represent a feasible staging approach in carefully selected patients with early-stage oral squamous cell carcinoma and clinically negative lymph nodes (cN0). The procedure demonstrated a high identification rate, low estimated radiation exposure, and successful detection of occult nodal metastases. However, the limited sample size and follow-up duration warrant cautious interpretation of the oncologic outcomes, and further prospective studies are required before the role of SLNB relative to elective neck dissection can be established.

## Introduction

Accurate assessment of locoregional lymph node involvement is a key component of oncologic management. Sentinel lymph node biopsy (SLNB) was developed to reduce surgical morbidity while maintaining oncologic safety. SLNB using technetium-99m is now a well-established procedure in the management of melanoma and non-melanoma skin cancers (1–3). Its application for oral squamous cell carcinoma (OSCC) remains less widespread, however, several centers have reported encouraging results using this technique (4, 5). Accordingly, current German guidelines acknowledge the role of SLNB in the management of selected head and neck malignancies (6–8).

Against this background, sentinel lymph node biopsy (SLNB) has emerged as a minimally invasive technique for assessing regional lymph node status in patients with oral cavity malignancies. The procedure allows targeted evaluation of the first draining lymph nodes and represents a minimally invasive approach for regional lymph node staging that may help reduce the need for more extensive surgical procedures. In this study, we evaluated the clinical use of SLNB using [99mTc]Tc-albumin nanocolloid in patients with oral squamous cell carcinoma treated at our institution. The primary aim was to assess oncologic safety and efficacy, including the detection of metastatic lymph nodes and oncologic outcomes during follow-up. Secondary endpoints included the quality of patient selection, evaluated by concordance between clinical and pathological TNM staging, as well as radiation exposure for patients and healthcare staff.

## Materials and methods

### Study design and ethical approval

This retrospective single-center study evaluated patients undergoing sentinel lymph node biopsy (SLNB) for oral squamous cell carcinoma. The study was approved by the Ethics Commission of the University of Witten-Herdecke (No. S-41/2024). All procedures were conducted in accordance with the principles of the Declaration of Helsinki.

### Patient selection and treatment workflow

The treatment process began with clinical staging and histological biopsy of the primary tumor. If the oral squamous cell carcinoma was clinically and radiologically classified as cT1-cT2 without evidence of lymph node metastasis (cN0), SLNB was considered during treatment planning. If additional surgical procedures, such as osteotomies, were required and a minimally invasive approach was not considered feasible, a neck dissection was performed instead. If SLNB was considered feasible, patients were informed about all treatment options, including both neck dissection and SLNB, and provided informed consent before surgery. In addition to tumor stage and radiological nodal status, patient-related factors such as overall medical condition and perioperative risk (ASA classification) were considered during treatment planning. Therefore, the decision to perform SLNB was individualized and based on anatomical feasibility, the anticipated duration of surgery, and patient fitness for more extensive cervical procedures. Patients with histologically confirmed sentinel lymph node metastasis were generally recommended to undergo completion neck dissection. However, the final treatment decision was individualized following patient-specific considerations.

### Radiotracer application and imaging

Patients scheduled for SLNB are referred to the Department of Nuclear Medicine. Radiotracer injection and imaging were performed approximately 24 hours before oncologic surgery. The radiotracer [99mTc]Tc-albumin nanocolloid was prepared immediately before application by nuclear medicine physicians according to established preparation protocols.

Intraoral injections were performed by an oral & maxillofacial surgeon (ÁB). Immediately after the radiotracer application, lymphatic drainage and tracer deposition in the head-neck-region were documented using planar scintigraphy on a gamma camera.

After approximately two hours, a low-dose SPECT-CT scan was performed, to further localize the sentinel lymph nodes. In addition, count rates above the injection site (radiotracer depot) and the SLNBs were measured and documented using a mobile gamma probe (Neoprobe^®^, Fa. Mammotome).

### Surgical procedure

To reduce personal exposure for the surgical team, the injected activity and timing of tracer application were calculated considering radioactive decay so that a maximum of 10 MBq remained in the patient at the time of surgery.

Before starting the surgery, the functionality of the gamma probe used was tested in accordance with German regulatory standards (DIN 6855-1). The surgical approach followed standard cervical incision. Skin incisions were planned along natural skin folds, and whenever possible a single incision was used to access one or more lymph node sites.

During surgery, the gamma probe was placed in a sterile sheath and used to identify the sentinel lymph nodes intraoperatively. Excised lymph nodes were also measured ex vivo with the gamma probe to confirm tracer uptake. The surgical field was subsequently re-examined with the probe to detect additional nodes until only minimal background activity is present. The excised lymph nodes were submitted for histopathological examination. Histological results were typically available within 4–5 days. If no metastatic involvement was detected (pN0), no further surgical treatment was required. In cases of nodal metastasis (pN+), a neck dissection was performed.

### Radiation dose assessment

Personal radiation exposure of the surgeon was not measured directly using personal dosimeters but was estimated using a point source approximation. Α dose rate coefficient of 0.0216 $\frac{\text{Gy}\cdot {\text{m}}^{2}}{\text{h}\cdot \text{GBq}}$ ς was applied in accordance with the German standard DIN 6844-3. The average distance between the surgeon and the radiation source was assumed to be 0.3 m. The radiation dose D  received by the surgeon was calculated according to the following equation:


$$D=0.0216\frac{\text{Gy}\cdot {\text{m}}^{2}}{\text{h}\cdot \text{GBq}}\cdot {\left(\frac{1}{0.3}\right)}^{2}\cdot t_{\text{surgery}}$$

where $t_{\text{surgery}}$ represents the duration of the surgical procedure.

### Follow-up

Patients underwent a standardized oncologic follow-up protocol. During the first two years after surgery, clinical examinations were performed every three months, and contrast-enhanced CT scans of the head and neck region were obtained every six months. Between the third and fifth year of follow-up, clinical examinations were performed every six months, and contrast-enhanced CT imaging was conducted annually.

### Data management and statistical analysis

Data were collected and managed using the REDCap electronic data capture system (9, 10). Demographic, clinical, and procedural data were entered into the study database and analyzed statistically using SPSS version 29.0 (IBM Corp., Armonk, NY, USA). Data distribution was assessed using the Kolmogorov–Smirnov test. Descriptive statistics were used for demographic and clinical variables. Survival and follow-up outcomes were evaluated using Kaplan–Meier analysis. Results are presented as descriptive statistics, tables, and graphical illustrations including boxplots and Kaplan–Meier curves.

## Results

A total of 16 patients were included in the study (mean age 70.9 ± 11.1 years). The cohort consisted of 11 male patients and 5 females. Baseline patient and tumor characteristics are summarized in **Table 1**.

**Table 1:** Clinicopathologic characteristics.

Variable	Value
Number of patients	16
Age (years), mean ± SD	70.9 ± 11.1
Sex	
Male	11 (68.8%)
Female	5 (31.2%)
Pathological T stage (pT)	
1	13 (81,3%)
2	1 (6.3%)
4a	2 (12.4%)
Pathological lymph node status (pN)	
pN0	13 (81.2%)
pN+	3 (18.8%)
Overall pathological UICC stage	
Stage I	11 (68.8%)
Stage II	0
Stage III	1 (6.2%)
Stage IV	4 (25%)

UICC, Union for International Cancer Control.

### Tumor characteristics

The study cohort included only patients with squamous cell carcinoma of the oral cavity. No other histological tumor types or primary sites were included in the analysis. The detailed anatomical distribution of oral cavity tumors is presented in **Table 2**, with the floor of the mouth and lateral border of tongue being the most common sites of occurrence, consisting 25% and 18.8% of the cases respectively.

**Table 2:** Localization of oral cavity malignancies.

Variable	Value
Buccal mucosa	1 (6.2%)
Floor of mouth	4 (25%)
Hard palate	2 (12.5%)
Lateral border of tongue	3 (18.8%)
Mandibular alveolar ridge	2 (12.5%)
Maxillary alveolar ridge	2 (12.5%)
Maxillary vestibulum	1 (6.2%)
Tongue tip	1 (6.2%)
Total	16 (100%)

### Clinical and pathological staging

Τumor localization together with the corresponding clinical and pathological staging for each patient included in the study are summarized in **Table 3**. Occult lymph node metastases were detected in three of the 16 patients (18.8%), corresponding to cases in which the pathological lymph node status was higher than the initial clinical staging. All positive sentinel lymph nodes contained macrometastatic disease (>2 mm), whereas no micrometastases or isolated tumor cells were identified. In addition, pathological examination revealed tumor upstaging (pT>cT) in one of the 16 cases (6.3%). Notably, the patient with tumor upstaging also demonstrated occult nodal metastasis, indicating concordant pathological upstaging of both the primary tumor and nodal status.

**Table 3:** Localization, clinical and pathological staging.

Localization	Clinical stage (cTcN)	Pathological stage (pTpN)
Maxillary vestibule	1 0	1 0
Floor of mouth	1 0	1 0
Lateral tongue	1 0	1 0
Maxillary alveolar ridge	1 0	1 0
Hard palate	1 0	1 0
Floor of mouth	1 0	2 2b
Lateral tongue	1 0	1 0
Buccal mucosa	1 0	1 0
Floor of mouth	1 0	1 0
Tongue tip	1 0	1 0
Mandibular alveolar ridge	1 0	1 1
Mandibular alveolar ridge	1 0	1 0
Maxillary alveolar ridge	4a 0	4a 0
Floor of mouth	1 0	1 2a
Lateral tongue	1 0	1 0
Hard palate	4a 0	4a 0

### Surgical and radiation parameters

The initially administered activity of the ^99m^Tc averaged 100.9 ± 2.1 MBq. The mean interval time between tracer administration and surgery was 26 h 03 min (range 23 h 31 min to 29 h 44 min). The mean duration of the surgery was 111.5 min, ranging from 57 min to 276 min.

**Figure 1:**
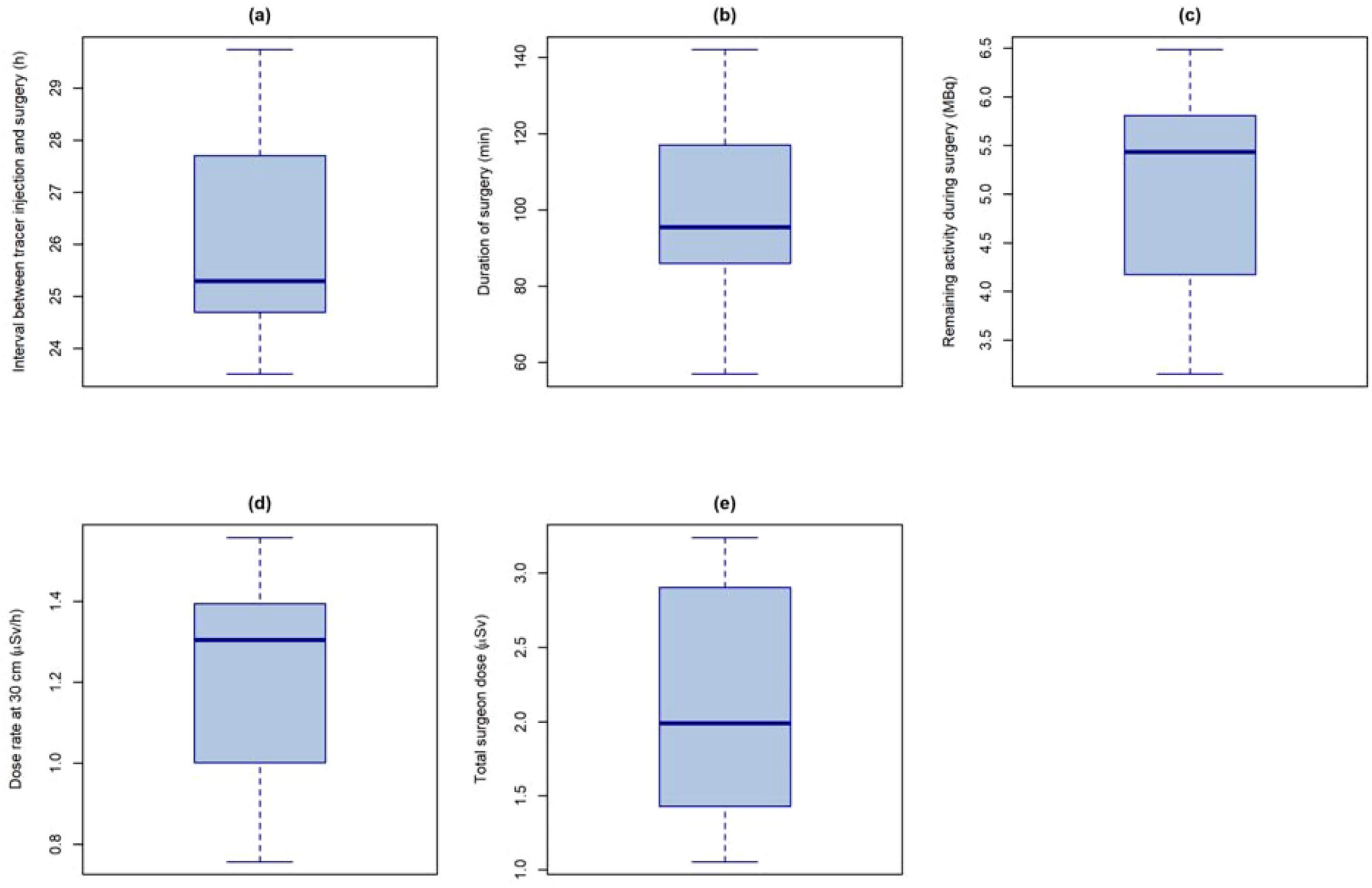
Boxplots illustrating surgical and radiation parameters: **(a)** time interval between radiotracer injection and start of surgery; **(b)** duration of surgery; **(c)** remaining technetium-99m activity during surgery; **(d)** dose rate measured at a distance of 30 cm from the radiation source during surgery; **(e)** total radiation dose to the surgeon measured at a distance of 30 cm.

During surgery, the remaining radioactive activity averaged 5.1 MBq (range 3.2 - 6.5 MBq). The mean dose rate measured at a distance of 30 cm from the source was 1.2 μSv/h (range 0.8–1.6 μSv/h), corresponding to a mean total radiation exposure of 2.3 μSv (range 1.1–5.7 μSv) for the surgeon. The corresponding distributions are illustrated in **Figures 1a–e**.

### Follow-up and survival

**Figure 2:**
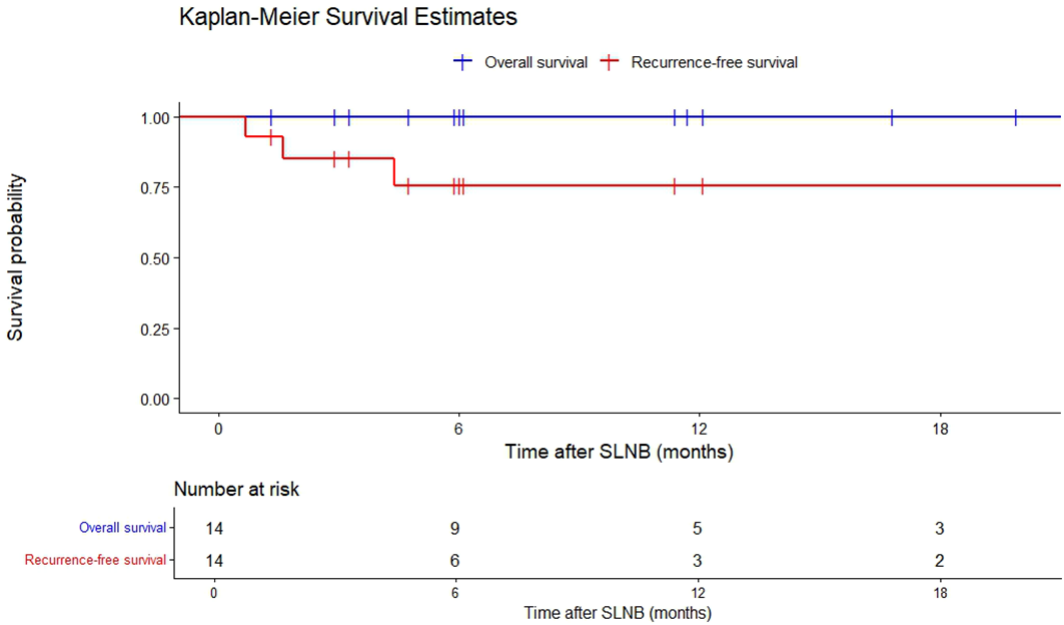
Kaplan–Meier overall survival curve of patients with oral squamous cell carcinoma undergoing sentinel lymph node biopsy during the follow-up period.

Overall survival during follow-up is presented in **Figure 2**. During the observation period, ranging from a minimum of 90 days to a maximum of 937 days, with a median follow-up of 266 days, all patients were alive at the time of database lock. No regional nodal recurrences were observed during follow-up. Furthermore, all patients with pathological lymph node metastases were correctly identified by SLNB, and no delayed nodal failures occurred among SLNB-negative patients.

## Discussion

The present study suggests that sentinel lymph node biopsy (SLNB) may represent a feasible and reliable staging approach in selected patients with oral squamous cell carcinoma. Occult lymph node metastases were detected in 18.8% of patients, while tumor upstaging (pT>cT) occurred in 6.3% of cases, highlighting limitations of preoperative clinical staging. Sentinel lymph nodes were successfully identified in all patients using combined SPECT-CT imaging and intraoperative gamma probe detection. In patients with positive sentinel lymph nodes, no additional metastatic lymph nodes were detected in subsequent neck dissection specimens. Furthermore, SLNB was associated with low estimate radiation exposure for both patients and surgical staff, while the applied tracer activity provided reliable intraoperative localization of sentinel lymph nodes. Although these findings support the use of SLNB as a minimally invasive staging approach in selected patients with early stage OSCC, the limited sample size and follow-up duration warrant cautious interpretation of the oncologic outcomes.

Compared with the available literature, our findings are largely consistent with previously reported data (11). Furthermore, studies using nanoparticle-based tracers have demonstrated comparable detection rates to those observed in our cohort (12). Reported positivity rates for SLNB for oral squamous cell carcinoma range between 4% and 21.9% (13), with our observed rate of 18.8%. Tumor size greater than 20 mm has been identified as a predictor of nodal metastasis, highlighting the importance of careful patient selection when considering SLNB (1, 4, 14–17). However, the optimal threshold for performing elective neck dissection remains uncertain, particularly in cT2 cN0 patients, while further multicenter studies are required to better define appropriate treatment strategies in this subgroup. Tumor-related factors may also play an important role in predicting nodal metastasis. Previous studies have identified tumor size, depth of invasion, and other pathological characteristics as important predictors of occult cervical metastasis, highlighting the importance of careful patient selection when considering SLNB (3, 16, 18, 19).

Radiation protection has become increasingly important, with progressively stricter limits for occupational exposure introduced over recent decades (20). German regulations, which are among the most stringent worldwide, define annual dose limits for surgical staff involved in SLNB of 1 mSv (effective dose), 15 mSv (eye lens), and 50 mSv (skin) (21). In our study, the radiation exposure to the surgeon ranged from 1.1 to 5.7 μSv at a distance of 30 cm from the radiation source, without considering any shielding between the patient and the surgeon. Assuming a tumor center with approximately 120 new patients per year, of whom 10–15 may be eligible for SLNB, the cumulative radiation exposure remains well below the established legal limits. These findings indicate that the applied activity of technetium-99m is sufficient for reliable detection while maintaining low radiation exposure for both patients and medical staff. Standard protective measures for personnel involved in tracer preparation and surgical procedures appear to be adequate. For safety reasons, clear documentation of the timing and application of the radioactive tracer is essential in all relevant patient records. To the best of our knowledge, comparable international data on occupational radiation exposure in this setting remain limited.

Despite the encouraging results, this study has several limitations. First, the relatively small sample size limits the generalizability of the findings and does not allow definitive conclusions to be drawn for all patient subgroups. Furthermore, the study population was highly selected, including only patients with early-stage disease and clinically negative lymph nodes (cN0). As treatment allocation was based on clinical judgement, anatomical feasibility, perioperative risk, and patient preference, a degree of selection bias cannot be excluded. Although no regional nodal recurrences or delayed nodal failures were observed during follow-up, the relatively limited follow-up duration and small cohort size preclude robust estimation of disease-free survival and the false-negative rate of SLNB. Lastly, although no additional metastatic lymph nodes were identified in cases with positive sentinel lymph nodes after neck dissection, the sample size is insufficient to draw firm conclusions regarding the reliability of SLNB in all cases. Careful preoperative staging and patient selection remain essential prerequisites for the safe and effective use of this approach.

## Conclusions

Sentinel lymph node biopsy (SLNB) may be a feasible method for staging carefully selected patients with early-stage oral squamous cell carcinoma and clinically negative lymph nodes (cN0). However, the role of SLNB in selected patients remains uncertain and requires further investigation. Although SLNB appears to be a less invasive staging approach, further prospective studies are needed before its role relative to elective neck dissection can be established.

## Data Availability

The raw data supporting the conclusions of this article will be made available by the authors, without undue reservation.

## References

[1] Civantos F Helmen ZM Bradley PJ Coca-Pelaz A De Bree R Guntinas-Lichius O . Lymph node metastases from non-melanoma skin cancer of the head and neck. Cancers. (2023) 15:4201. doi: 10.3390/cancers15174201 37686478 PMC10486745

[2] Mahieu R Donders DNV Dankbaar JW de Bree R de Keizer B . CT lymphography using Lipiodol® for sentinel lymph node biopsy in early-stage oral cancer. J Clin Med. (2022) 11:5129. doi: 10.3390/jcm11175129 36079061 PMC9456579

[3] Zhang Y Liu C Wang Z Zhu G Zhang Y Xu Y . Sentinel lymph node biopsy in head and neck cutaneous melanomas: A PRISMA-compliant systematic review and meta-analysis. Med (Baltimore). (2021) 100:e24284. doi: 10.1097/MD.0000000000024284 33592872 PMC7870248

[4] Bark R Kolev A Elliot A Piersiala K Näsman A Grybäck P . Sentinel node‐assisted neck dissection in advanced oral squamous cell carcinoma—A new protocol for staging and treatment. Cancer Med. (2023) 12:12524–34. doi: 10.1002/cam4.5966 37084007 PMC10278494

[5] De Bree R de Keizer B Civantos F Takes RP Radboud . What is the role of sentinel lymph node biopsy in the management of oral cancer in 2020? Eur Arch Otorhinolaryngol. (2021) 278:3181–91. doi: 10.1007/s00405-020-06538-y 33369691 PMC8328894

[6] Leitlinienprogramm Onkologie (Deutsche Krebsgesellschaft, Deutsche Krebshilfe, AWMF) (2022). S3-Leitlinie Aktinische Keratose und Plattenepithelkarzinom der Haut (Langversion 2.0). (Berlin: Leitlinienprogramm Onkologie).

[7] Leitlinienprogramm Onkologie (Deutsche Krebsgesellschaft, Deutsche Krebshilfe, AWMF) (2020). S3-Leitlinie: Diagnostik, Therapie und Nachsorge des Melanoms (Langversion 3.3). (Berlin: Leitlinienprogramm Onkologie).

[8] Leitlinienprogramm Onkologie (Deutsche Krebsgesellschaft, Deutsche Krebshilfe, AWMF) (2021). S3-Leitlinie Diagnostik und Therapie des Mundhöhlenkarzinoms (Langversion 3.0). (Berlin: Leitlinienprogramm Onkologie).

[9] Harris PA Taylor R Minor BL Elliott V Fernandez M O'Neal L . The REDCap consortium: Building an international community of software platform partners. J BioMed Inform. (2019) 95:103208. doi: 10.1016/j.jbi.2019.103208 31078660 PMC7254481

[10] Harris PA Taylor R Thielke R Payne J Gonzalez N Conde JG . Research electronic data capture (REDCap)—A metadata-driven methodology and workflow process for providing translational research informatics support. J BioMed Inform. (2009) 42:377–81. doi: 10.1016/j.jbi.2008.08.010 18929686 PMC2700030

[11] Hirshoren N Abd El Qadir N Weinberger JM Eliashar R Ben-Haim S . Sentinel lymph node identification in cutaneous head & neck cancer - lymphoscintigraphy late phase. Laryngoscope. (2022) 132:2164–8. doi: 10.1002/lary.30076 35199860 PMC9790693

[12] Zanoni DK Stambuk HE Madajewski B Montero PH Matsuura D Busam KJ . Use of ultrasmall core-shell fluorescent silica nanoparticles for image-guided sentinel lymph node biopsy in head and neck melanoma: A nonrandomized clinical trial. JAMA Netw Open. (2021) 4:e211936. doi: 10.1001/jamanetworkopen.2021.1936 33734415 PMC7974643

[13] Spyropoulou GA Mpalaris V Pervana S Trakatelli M Foroglou P Milothridis P . Prospective randomized study on the use of sentinel node biopsy for high-risk cutaneous squamous cell carcinomas of the head and neck. Plast Reconstr Surg - Glob Open. (2024) 12:e6092. doi: 10.1097/GOX.0000000000006092 39188963 PMC11346874

[14] Abdul-Razak M Mwagiru D Veness M Wong E Pang T Morgan G . Does sentinel lymph node biopsy accurately stage the clinically negative neck in early oral cavity squamous cell carcinoma? J Oral Maxillofac Surg. (2022) 80:1134–42. doi: 10.1016/j.joms.2022.02.006 35304106

[15] den Toom IJ Boeve K Lobeek D Bloemena E Donswijk ML de Keizer B . Elective neck dissection or sentinel lymph node biopsy in early stage oral cavity cancer patients: The Dutch experience. Cancers. (2020) 12:1783. doi: 10.3390/cancers12071783 32635357 PMC7407164

[16] Han AY John M . Predictors of nodal metastasis in cutaneous head and neck cancers. Curr Oncol Rep. (2022) 24:1145–52. doi: 10.1007/s11912-022-01249-5 35394247 PMC9468084

[17] Vuity D McMahon J Hislop S McCaul J Wales C Ansell M . Sentinel lymph node biopsy for early oral cancer – accuracy and considerations in patient selection. Br J Oral Maxillofac Surg. (2022) 60:830–6. doi: 10.1016/j.bjoms.2021.12.058 35331563

[18] Ionna F Pavone E Aversa C Maffia F Spinelli R Carraturo E . Sentinel lymph node biopsy (SLNB) for early-stage head and neck squamous-cell carcinoma of the tongue: Twenty years of experience at I.N.T. “G.Pascale. Cancers. (2024) 16:1153. doi: 10.3390/cancers16061153 38539488 PMC10969103

[19] Javidiparsijani S Brickman A Lin DM Rohra P Ghai R Bitterman P . Is regional lymph node metastasis of head and neck paraganglioma a sign of aggressive clinical behavior: A clinical/pathologic review. Ear Nose Throat J. (2021) 100:447–53. doi: 10.1177/0145561319863373 31566000

[20] Boice J Dauer LT Kase KR Mettler FA Vetter RJ . Evolution of radiation protection for medical workers. Br J Radiol. (2020) 93:20200282. doi: 10.1259/bjr.20200282 32496817 PMC7446021

[21] Germany, Federal Government (Bundesregierung) . Verordnung zur weiteren Modernisierung des Strahlenschutzrechts. Bundesgesetzblatt Teil I. (2018) 2034. (Köln: Bundesanzeiger Verlag GmbH).

